# Performance-based instrument to assess functional capacity in community-dwelling older adults

**DOI:** 10.1590/1980-57642018dn13-040004

**Published:** 2019

**Authors:** Ana Cláudia Becattini-Oliveira, Leonardo Cardoso Portela Câmara, Douglas de Farias Dutra, Antonia de Azevedo Falcão Sigrist, Helenice Charchat-Fichman

**Affiliations:** 1 Psychology Department, Pontifícia Universidade Católica do Rio de Janeiro (PUC-Rio), Rio de Janeiro, RJ, Brazil.; 2 Psychology Institute, Universidade Federal do Rio de Janeiro (UFRJ), Rio de Janeiro, RJ, Brazil.

**Keywords:** functional capacity, cognition, aged, dementia., capacidade funcional, cognição, idosos, demência.

## Abstract

Functional capacity (FC) is a mediator between neuropsychological functions and real-world functioning, but there is a lack of evidence of its correlation in community-dwelling older adults. Objective: The study aim was to determine the FC level of community-dwelling older adults using the UCSD Performance-based Skills Assessment (UPSA) and to evaluate correlation with cognitive screening tests. Methods: Senior center participants were selected according to inclusion criteria: Portuguese fluency, age ≥60 years and self-reported independent living. The subject exclusion criteria were: dementia or other DSM-5 diagnoses, suicidal ideation or intent, non-completion of assessment battery, enrollment in another psychosocial intervention or pharmacotherapy study. FC level was determined by the UPSA, brief UPSA (UPSA-B) and Instrumental Activities of Daily Living scale (IADL’s). The Mini-Mental State Examination (MMSE), Memory of Figure Test (MFT), Verbal Fluency Test (VFT) and Clock Drawing Test (CDT) were used for cognitive assessment. A total of 35 subjects that had a mean age of 72 years, were predominantly females(88.6%) and had mean education level of 11.25 years were evaluated. Results: Mean UPSA and UPSA-B scores were 78.5 and 70, respectively. A statistically significant correlation was observed between the UPSA and IADL, MMSE and VFT. Conclusion: The UPSA serves as a screening assessment of FC in community-dwelling older adults, showing a positive correlation with cognitive screening tests.

## RESUMO

Capacidade Funcional (CF) é um mediador entre as funções neuropsicológica e a funcionalidade no mundo real, mas existe uma falta de evidências da sua correlação em idosos vivendo em comunidade. Objetivo: O objetivo foi determinar a CF de idosos da comunidade utilizando o *UCSD Performance-based Skills Assessment* (UPSA) e avaliar sua correlação com testes de rastreio cognitivo. Métodos: Participantes de casas de convivência foram selecionados conforme critérios de inclusão: fluência no Português; 60 ou mais anos; auto relato de independência. Critérios de exclusão foram: demência ou outro diagnóstico do DSM-5; ideação suicida ou tentativa, não adesão a ao estudo; envolvimento em estudo farmacológicos ou intervenção psicoterápica. A CF foi determinado pelo UPSA, UPSA abreviado (UPSA-B) e escala Instrumental de Atividade de Vida Diária (AIVD). O Mini-Exame do Estado Mental (MEEM), Teste de Memória de Figuras (TMF), Teste de Fluência Verbal (TFV) e Teste do Desenho do Relógio (TDR) foram utilizados no rastreio cognitivo. Um total de 35 sujeitos com idade média de 72 anos, predominantemente do sexo feminino (88,6%) e escolaridade média de 11,25 anos foram avaliados. Resultados: A pontuação média do UPSA e UPSA-B foram 78,5 e 70, respectivamente. Uma correlação foi observada entre o UPSA e AIVD, MEEM e TFV. Conclusão: O UPSA é um teste de rastreamento da CF de idosos da comunidade apresentando correlação positiva com testes de rastreio cognitivo.

The world population is rapidly ageing with major consequences for health systems and their workforce. In 2015, the world population over 60 years old numbered 900 million and is set to reach a total of 2.1 billion in 2050.^1^ It is also expected that two-thirds of this population will be living in low-income countries.^2^ The rapid increase in non-communicable diseases such as dementia has a high economic burden. In 2015, a total of 46.8 million cases of dementia were diagnosed worldwide with an estimated cost of 818 billion of dollars.^3^ Projections indicate that the number of cases could double within just twenty years, affecting more than 90 million. The early detection of cognitive impairment in community-dwelling older adults is still a challenge in this field. The prevention of cognitive impairment may delay progression to stages of high dependence, thus reducing the economic impact on the health system.

Functional capacity (FC) is defined as the minimum set of abilities that are essential to function independently in the community and is a known mediator between neuropsychological ability and real-world functioning.^4^^-^ 6 Studies involving psychiatric patients have demonstrated that global neuropsychological performance is related to performance across multiple real-world functional domains, including participation in community activities, interpersonal functioning, and work skills, though this relationship was largely mediated by FC scores.^7^ These studies suggest that patients with better overall neurocognitive skills are likely to have greater FC and subsequent better outcomes. Since FC was adopted as a reference marker in psychiatric conditions such as schizophrenia, improvement in FC outcome has been required as a co-primary measure in clinical trials.^8^^,^ 9 FC measurements have also been required in clinical trials involving Alzheimer’s disease(AD),^10^^,^ 11 as one of the treatment targets is to delay the progression of AD to stages with higher levels of dependency. 

FC measurement techniques have strengths and weakness,^12^^,^ 13 particularly in community-dwelling individuals. In general, these techniques are based on abilities/skills to function in the real world such as communication skills and finance management. Most techniques have focused on FC measurement via self-reporting, proxy ratings, clinician ratings, and/or direct observation of patient’s behavior in their own environment. Self-reporting FC scores may be affected by depression, lack of self-awareness, cognitive and emotional functioning, and social judgments. Furthermore, functional scores based on a patient’s behavior in an interview setting may not relate directly to capabilities in a range of domains in the outside world. Proxy ratings may be unreliable, as they depend on the observational ability and level of engagement of the informant. Clinician ratings are often based on brief contact with the patient and focus on behaviors that may or may not be related to the patient’s ability to function in the real world. Although assessment of behaviors through direct observation of patients in their own environment during long periods can provide representative data,it is likely to be extremely expensive and labor-intensive. Performance-based assessment techniques have proven to be a viable alternative when used in psychiatric patients living in the community, demonstrating improvements in FC measurement.^14^

The UCSD Performance-Based Skills Assessment (UPSA)^13^ directly assesses FC using standard role-playing to evaluate performance in five domains: Comprehension and Planning (e.g., organizing trips), Finance (e.g., paying bills), Communication (e.g., using the telephone for an appointment), Transportation (e.g., public transportation) and Household Chores (e.g., planning shopping). The total scores range from 0 to 100 and the test takes about 30 minutes to complete,though there are shorter versions of the UPSA based on 4 (UPSA-2) and 2 (UPSA-B) domains.^7^^,^ 15 ^,^ 16 The UPSA was originally developed in English to evaluate schizophrenia patients living in the community. It was based on the self-report Instrumental Activity of Daily Living (IADL) and had concordant validity with the *Direct Assessment of Functional Status* (DAFS) and *Mattis Dementia Rating Scale* (MDRS).^6^^,^ 13 Since 2001 it has been culturally adapted to 8 different languages and employed in studies conducted in 17 countries.^17^ These studies involved FC measurement in a variety of neuropsychiatric conditions such as Bipolar Disorder, Post-Traumatic Stress Disorder, Mild Cognitive Impairment (MCI) and AD.^18^^-^ 21 More recently, UPSA was also employed in studies involving patients with HIV infection.^22^ A systematic review conducted by our group to identify the psychometric properties of the UPSA^17^ extracted, summarized and analyzed data from 41 studies involving 8,782 community-dwelling individuals. Although few studies reported sensitivity and specificity, moderate-to-strong evidence of construct validity and test-retest reliability was found. The UPSA showed good psychometric properties in its shorter versions, including those with cultural adaptations. The UPSA also strongly correlated with the WAIS-III, a cognitive battery based on classical neuropsychological paradigms that has robust psychometric properties.^23^

Early detection of functional decline in community-dwelling older adults is still a challenge, as they constitute an extremely heterogeneous population with many confounding variables such as education and other lifestyle factors that may influence cognitive reserve.^24^ The aim of this study was to determinate the functional capacity level of community-dwelling older adults using the UCSD Performance-based Skills Assessment (UPSA) and the UPSA-B and to evaluate their correlation with cognitive screening instruments. 

## METHODS

### Sample selection

This study was part of a larger study involving participants of senior centers administered by the city council of Rio de Janeiro^25^ and was approved by the Research Ethics Committee (opinion no. 965.264). The selection process was based on an interview conducted by a geriatrician of the research team according to the attendance capacity of the two senior centers where the research was conducted. The inclusion criteria were: Portuguese fluency, minimum age of 60 years and independent living ability. These criteria were endorsed by the requirements for senior center use (absence of a caregiver and regular frequency for their activities). Participants were excluded based on the following criteria: diagnosis of dementia or another DSM-5 diagnosis,^26^ suicidal ideation or intent, inability to complete the assessment battery (sensory or motor impairment), and enrollment in another psychosocial intervention and/or pharmacotherapy study. Participants that met the selection criteria provided informed written consent for entry to the study. 

From a total of 40 interviewed subjects, 5 were excluded (refused to participate, did not meet selection criteria or did not complete the research protocol). The sample of 35 subjects comprised predominantly females (88%), individuals with a mean age of 72.26 (SD=5.98) years and mean educational level of 11.26 (SD=4.63) years. The mean number of chronic diseases was 2.71 (SD=1.40; ranging from 0 to 5), with a mean number of regularly used medications of 2.85 (SD=1.90; ranging from 0 to 7). Table 1 summarizes the sample characteristics.

**Table 1:** Descriptive statistics of the cognitive and functioning measures.

	Mean (SD)	Median (Quartile range)
Age	72.26 (5.98)	71.00 (69-78)
Educational Level	11.26 (4.63)	12.00 (9-17)
Diseases	2.71 (1.41)	3.00 (2-4)
Medicines	2.85 (1.91)	2.00 (1-4.25)
MFT Naming	9.91 (.28)	10.00 (10-10)
MFT Incidental 1	5.71 (1.53)	5.00 (5-7)
MFT Incidental 2	8.46 (1.46)	9.00 (8-10)
MFT Incidental 3	8.89 (1.59)	10.00 (8-10)
MFT Episodic	8.34 (1.61)	9.00 (7-10)
MFT Recognition	9.40 (2.35)	10.00 (10-10)
CDT	5.91 (2.15)	5.00 (5-8)
VFT	18.03 (8.03)	18.00 (13-21)
MMSE	26.29 (3.34)	27.00 (25-28)
IAVD	20.06 (1.14)	20.00 (20-21)
UPSA Planning	17.12 (2.35)	17.04 (16.30-19.26)
UPSA Finance	15.26 (5.25)	18.00 (12-20)
UPSA Communication	12.76 (4.42)	13.33 (8.89-15.56)
UPSA Transportation	15.24 (3.82)	16.67 (13.34-16.67)
UPSA Household Chores	18.14 (5.16)	20.00 (20-20)
UPSA Total score	78.52 (15.52)	81.70 (72.22-89.26)
UPSA-B	70.05 (21.46)	77.78 (61.67-84.44)

MFT: Memory of Figure Test; CDT: Clock Drawing Test; VFT: Verbal Fluency Test; MMSE-30: Mini Mental State Examination; UPSA: UCSD Performance-based Skill Assessment; UPSA-B: UCSD Performance-based Skill Assessment Brief version.

### Data collection

The subjects were evaluated in one session meeting of 60-90 minutes duration conducted by a previously trained psychologist from the research team. Demographic characteristics and health status information were collected from the geriatrician’s interview during the selection process.

The UPSA^13^^,^ 27 and Instrumental Activity of Daily Living (IADL)^28^ were employed to assess FC measures. The UPSA-B^16^^,^ 27 scores were calculated according to the communication and finance domains of the original version. 

The Brief Cognitive Screening Battery (BCSB)^29^^-^ 31 was employed to assess cognitive measures. The BCSB instruments were applied in the following sequence: Mini-Mental State Examination (MMSE),^32^^,^ 33 Memory of Figure Test (MFT),^29^^,^ 34 Verbal Fluency Test - animals category (VFT)^35^^,^ 36 and Clock Drawing Test (CDT).^29^^,^ 37 ^,^ 38 For this study, cut-off points were adopted according to previous studies and sample characteristics (age and level of education).^39^

### Statistical analysis

Data analysis was performed using the IBM SPSS software version 22. Skewness and kurtosis values were measured to assess the normality of the data distribution. Kendall’s Tau-b, a non-parametric correlation analysis, was used to measure the association between variables. Kendall’s Tau-b was preferred over Pearson’s correlation due to the greater accuracy of Kendall’s hypothesis test in smaller sample sizes.^40^

Hierarchical regression models were used to test associations between cognitive tests and functional capacity, while controlling for demographic variables. A two-step approach was used. On the first step sex, age and years of education were entered as predictors into the model. The male category was the reference for the sex variable. Age and years of education were measured as continuous variables. The overall fit (*F* statistic) and explained variance (adjusted R²) of the first step were registered. On the second step, the MFT (episodic memory trial), CDT, VFT and MMSE scores were introduced into the model. Changes in fit and explained variance from the first step to the second step were reported, as well as the overall fit and explained variance of the second step model. Both the UPSA and UPSA-B were used as dependent variables. As regression is a parametric analysis, bootstrapped confidence intervals were used to estimate the precision and significance of the predictors. 

The sample was divided into healthy and cognitively-impaired participants based on performance on the MMSE. Performance was categorized based on previously published data of 470 elderly residents of Rio de Janeiro on the BCSB, stratified by years of education. Participants were categorized as ‘cognitively impaired’ when their score on the MMSE was one or more standard deviations below the mean. The Functional Capacity and IADL scores of the two groups were compared with the Mann-Whitney U-test.

## RESULTS

Table 1 displays descriptive statistics for cognitive and functional capacity measures. Mean scores on cognitive tests are similar to previously published data. A non-parametric estimate, Kendall’s Tau-b, was employed to test variables with asymmetric distributions (Table 2). Age demonstrated negative correlation with the VFT, UPSA total score, UPSA-B and UPSA Communication and Transportation domains. Level of education correlated positively with the MMSE, VFT, CDT, UPSA total score, UPSA Finance and Transportation domains and the UPSA-B. The second (incidental memory) and fifth (episodic memory) steps of the MFT correlated positively with UPSA total score, and UPSA Finances and Transportation domains. The UPSA and the UPSA-B showed a positive moderate correlation with the MMSE, VFT, and CDT. The IADL correlated positively with UPSA total score and UPSA Comprehension/Planning and Finances domains.

**Table 2:** Non-parametric correlations (Kendall's tau-b) between cognitive and functioning measures.

	1	2	3	4	5	6	7	8	9	10	11	12	13	14	15	16	17	18	19
1. Age	1.000																		
2. Years of education	-0.126	1.000																	
3. MFT Naming	-0.086	0.304	1.000																
4. MFT Incidental 1	-0.196	0.119	0.197	1.000															
5. MFT Incidental 2	-0.229	0.121	0.108	0.643**	1.000														
6. MFT Incidental 3	-0.223	0.235	0.162	0.512**	0.642**	1.000													
7. MFT Episodic	-0.143	0.221	0.217	0.559**	0.742**	0.666**	1.000												
8. MFT Recognition	-0.188	-0.033	-0.093	0.014	0.157	0.331*	0.252	1.000											
9. CDT	-0.095	0.333*	0.397**	0.099	0.155	0.173	0.165	0.116	1.000										
10. VFT	-0.269*	0.363**	0.120	0.197	0.339**	0.213	0.297*	0.081	0.454**	1.000									
11. MMSE-30	-0.227	0.450**	0.275	0.300*	0.357**	0.337*	0.288*	0.127	0.371**	0.411**	1.000								
12. Lawton	-0.055	0.222	-0.056	0.091	0.234	0.261	0.128	-0.155	0.134	0.106	0.153	1.000							
13. Planning	-0.239	0.175	0.272	0.068	0.181	0.199	0.263*	0.026	0.278*	0.117	0.257*	0.311*	1.000						
14. Finances	-0.227	0.344*	0.179	0.219	0.280*	0.190	0.272*	0.100	0.396**	0.472**	0.486**	0.312*	0.354**	1.000					
15. Communication	-0.333**	0.228	0.090	0.210	0.161	0.192	0.197	0.226	0.235	0.256*	0.350**	0.148	0.240	0.403**	1.000				
16. Transportation	-0.288*	0.411**	0.199	0.242	0.320*	0.253	0.316*	0.115	0.250	0.345**	0.467**	0.146	0.274*	0.534**	0.388**	1.000			
17. Household	-0.229	0.241	0.187	-0.125	-0.040	-0.142	-0.085	0.193	0.448**	0.190	0.232	0.035	0.188	0.186	0.189	0.212	1.000		
18. UPSA Total	-0.365**	0.326*	0.176	0.216	0.311*	0.241	0.279*	0.241	0.419**	0.400**	0.520**	0.265*	0.421**	0.721**	0.570**	0.638**	0.359**	1.000	
19. UPSA-B Total	-0.328**	0.306*	0.161	0.224	0.249	0.212	0.261*	0.184	0.363**	0.378**	0.465**	0.263*	0.392**	0.683**	0.765**	0.518**	0.188	0.769**	1.000

* *p*<0.05;

** *p*<0.01;

MFT: Memory of Figure Test; CDT: Clock Drawing Test; VFT: Verbal Fluency Test; MMSE-30: Mini Mental State Examination; UPSA: UCSD Performance-based Skill Assessment; UPSA-B: UCSD Performance-based Skill Assessment Brief version

The first step of the regression model (age, sex and years of education)explained 39.9% of the variance of the UPSA total score (*F* (3, 31)=8.51, *p*<0.01). Younger participants scored significantly higher on the UPSA. Participants who spent more years in school also performed significantly better on the UPSA. The women had a mean score that was 3.5 points lower than the men, but this difference was not statistically significant (Table 3). The addition of the MFT, CDT, VFT and MMSE in the second step of the regression improved the model quality (*F_change_* (4, 27)=3.30, *p*<0.05) and increased the explained variance by 13.7%.The second step model explained, in total, 53.6% of variance (*F* (7, 27)=6.61, *p*<0.01). Age remained a statistically significant predictor, but years of education did not (*B*=0.86, 95% CI [-0.22, 1.89], β=0.25). The VFT was negatively associated with the UPSA total score, while all other cognitive variables were positively associated with the UPSA (Table 3). The CDT was the strongest and only statistically significant cognitive predictor (*B*=2.65, 95% CI [0.07, 5.24], β=0.37). The MMSE, MFT and VFT were weakly correlated with the UPSA total score (Table 3).

**Table 3:** Regression models with demographic and cognitive variables predicting UPSA and UPSA-B.

		UPSA					UPSA-B			
		B	95% Confidence Interval		b		B	95% Confidence Interval		b
			Lower	Upper				Lower	Upper	
**Block 1**	Intercept	125.339	86.068	166.322			127.382	67.536	181.926	
	Age	-0.878*	-1.515	-0.304	-0.338		-1.090	-1.862	-0.251	-0.304
	Sex (Female)	-3.507	-16.751	6.699	-0.073		-1.130	-12.978	12.611	-0.017
	Education	1.753**	0.543	2.998	0.523		1.992*	0.424	3.316	0.430
**Block 2**	Intercept	78.003	27.105	114.803			45.540	-50.699	99.088	
	Age	-0.743*	-1.392	-0.062	-0.286		-0.772	-1.952	0.803	-0.215
	Sex (Female)	-9.359	-26.650	5.296	-0.195		-6.625	-28.276	15.799	-0.100
	Education	0.856	-0.221	1.894	0.255		0.577	-0.989	1.702	0.124
	MFT Episodic	0.929	-2.303	5.313	0.096		0.342	-4.970	7.017	0.026
	CDT	2.654*	0.066	5.244	0.367		2.443	-2.347	5.866	0.244
	VFT	-0.097	-0.806	0.519	-0.050		0.233	-0.655	1.402	0.087
	MMSE-30	1.185	-0.633	4.092	0.255		2.213	-0.849	7.636	0.344

* *p* < 0.05;

** *p* < 0.01;

MFT: Memory of Figure Test; CDT: Clock Drawing Test; VFT: Verbal Fluency Test; MMSE-30: Mini Mental State Examination; UPSA: UCSD Performance-based Skill Assessment; UPSA-B: UCSD Performance-based Skill Assessment Brief version

In relation to the UPSA-B, the first step of the regression explained 26.4% of variance (*F* (3, 31)=5.07, *p*<0.01). Participants with more years of education performed significantly better on the UPSA-B. Age was not significantly related to the UPSA-B. The women had a mean score that was 1.13 points lower than the men, but this difference was not statistically significant. The addition of cognitive variables in the second step of the regression significantly increased model quality (*F_change_* (4, 27)=2.87, *p*<0.05) and explained an additional 14.3% of UPSA-B variance. The second step model explained, in total, 40.7% of UPSA-B variance (*F* (7, 27)=4.33, *p*<0.01). Years of education was no longer a significant predictor in the second step of the regression (*B*=0.58, 95% CI [-0.99, 1.70], β=0.12). All cognitive variables added on the second step were not statistically significant predictors, but were all positively correlated with the UPSA-B.

A final analysis was conducted using the Mann-Whitney U to test the difference in functional capacity and IADL between cognitively-impaired and cognitively-normal subjects. The group with low MMSE score had significantly lower UPSA (*U*=32.00, *p*<0.05; effect size *r*=0.34) and UPSA-B Total (*U*=32.50; *p*<0.05; effect size *r*=0.34) scores. However, the IADL scores did not differ significantly (U=56.50; *p*=0.35; effect size *r*=0.16).

## DISCUSSION

This study involved community-dwelling, highly educated older adults that were regularly engaged in social and physical activities. The subjects had a mean of 2.71 chronic diseases, 2.85 medications and low rates of alcohol and tobacco use. Previous studies demonstrated that, in 85% of cases, chronic diseases were the main cause of disability in those over 60 years old.^41^^,^ 42 The relation between chronic disease and health status was also explored in a study involving 504 older adults living in their own home environment.^43^ The authors observed that chronic disease negatively affected functional performance and increased hospitalization rates, reducing independence in everyday functioning. In the present study, the mean IADL score was 20 and mean UPSA and UPSA-B scores were 78.5 and 70.0, respectively, suggesting an independent profile for the study sample. The subjects not only lived in their own environment, but also required little or no supervision. Moreover, they did not have a family member nearby all the time and some of them lived alone. 

The geriatric assessment was developed to attend a need for a specific approach to the inpatient care of old adults.^44^^,^ 45 It has since been widely employed in a variety of settings in an attempt to establish the individual’s health conditions and develop specific, tailored care plans. FC is a relevant part of this assessment, providing an evaluation of the individual’s ability to live independently in the community. Nevertheless, FC assessment has been a challenge in community-dwelling older adults. It involves the interaction of physical and cognitive abilities with each other, as well as with the environment. In general, IADL is the gold-standard instrument employed to determine the ability to interact with the environment. In this study, an IADL score of 20 out of 21 (maximum score possible) was observed in 80% of the sample, suggesting a ceiling effect that was not seen in the UPSA. Previous studies involving larger samples with Mild Cognitive Impairment and early stages of Alzheimer disease also observed a discrepancy between UPSA and IADL measurements.^14^^,^ 21 These findings suggested inability of the IADL to identify functional capacity impairment in community-dwelling older adults.

The detection of early stages of dementia in community-dwelling old adults is still a challenge for many professionals.^10^ In general, it requires the individual’s perception to report a decline in cognitive abilities, the health professional’s experience and neuropsychological evaluation, which is not always available. The cognitive screening tests are also affected by educational level, requiring the adoption of different cut-off points according to the individual’s characteristics.^29^^,^ 31 ^,^ 46 The variable presentation patterns and speed of progression are also obstacles to the early detection of dementia. In previous studies, the UPSA and UPSA-B also demonstrated a strong-to-moderate convergent validity with cognitive measurements and known-group validity.^17^ As a screening tool, the UPSA-B has some advantages in comparison to its original version, as it requires less time to complete, almost no training, and uses accessible materials (a telephone, some notes and coins).^16^^,^ 47 The psychometric properties and ease of application of the UPSA-B as a screening tool could make it a useful instrument for detecting early stages of dementia, independent of the individual’s characteristics or professional experience of the interviewer, but more studies are necessary.

This study showed a stronger correlation between the UPSA and educational level than between the UPSA and age. This finding can be partially explained by the hypothesis of cognitive reserve that attributes higher cognitive abilities to education.^24^ The Lothian Birth Cohorts of 1936 evaluated data from 1091 subjects and identified that longer schooling was a significant predictor of higher scores on a latent general cognitive factor at age 70, independently of childhood IQ score.^48^ The study also identified that, although stimulating environments may help preserve cognitive ability in later life, individual differences in prior cognitive ability may also contribute to engagement in complex and intellectually stimulating activities.^49^ In general, education affects an individual’s ability to develop new strategies to manage the constraints imposed by the ageing process. For example, a person can use a phone book more frequently to look up telephone numbers that were previously known by heart when confronted with a memory impairment. This discrete compensatory strategy will not be noted,especially if this person is not engaged in labour activities that requires some degree of productivity. One must also consider the effect of time interval on the evolution of age-related changes and diseases in the acquisition of adapted behaviours. All these factors can be obstacles in FC measurements based on self-report and proxy rating.

In this study, some subjects had scores below the cut-off point for screening tests, suggesting the presence of cognitive impairment. This aspect should be considered in the overall approach used with senior center participants, when defining the physical and cognitive stimuli applied as part of an active ageing program. A brief systematic assessment, performed on a regular basis, would help detect the early stages of cognitive impairment, as well as the heterogeneity in cognitive profile for the senior center population. The observed differences in cognitive levels could be used to support the design and adaptation of specific activities for the senior center.

One limitation of this study was the small sample size. This aspect should be considered before proposing wider generalization of the results to the Brazilian population as a whole. Nevertheless, this initial study suggests greater UPSA validity as an FC measure of community-dwelling older adults than the IADL. Further investigations involving a more representative sample size could provide normative data for the UPSA. It could also examine the UPSA predictive value for cognitive impairment in older adults with high education. This investigation could provide valuable information about the applicability of the UPSA as a screening tool for health professionals.

In conclusion, the IADL questionnaire did not perform well in a sample of community-dwelling older adults, as evidenced by its ceiling effect. By contrast, the UPSA and UPSA-B detected a wider range of variation in functional capacity. Additionally, the UPSA and UPSA-B showed a close relationship with cognitive screening tests, even when age and education were controlled. For the screening of functional impairment in this population, a performance-based evaluation seems to be more adequate.
